# Chronic lymphocytic leukemia treatment algorithm 2018

**DOI:** 10.1038/s41408-018-0131-2

**Published:** 2018-10-03

**Authors:** Sameer A. Parikh

**Affiliations:** Division of Hematology, Mayo Clinic, Rochester, MN USA

## Abstract

The treatment landscape for patients with chronic lymphocytic leukemia (CLL) has changed considerably with the introduction of very effective oral targeted therapies (such as ibrutinib, idelalisib, and venetoclax), and next-generation anti-CD20 monoclonal antibodies (such as obinutuzumab). These agents lead to improved outcomes in CLL, even among patients with high-risk features, such as del17p13 or *TP53* mutation and unmutated immunoglobulin heavy chain (*IGHV*) genes. Each of these treatments is associated with a unique toxicity profile; in the absence of randomized data, the choice of one type of treatment over another depends on the co-morbidities of the patient. Chemoimmunotherapy still plays an important role in the management of previously untreated CLL patients, particularly among young fit patients who have standard risk FISH profile and mutated *IGHV* genes. Richter’s transformation of CLL remains a difficult complication to treat, although therapy with programmed death 1 inhibitors such as pembrolizumab and nivolumab has shown impressive responses in a subset of patients. Our ability to risk stratify CLL patients continues to evolve; the CLL-International Prognostic Index (CLL-IPI) is the best validated tool in predicting time to first therapy among previously untreated patients. This review summarizes the current approach to risk stratification and management of CLL patients.

## Introduction

The 2008 World Health Organization classification defines chronic lymphocytic leukemia (CLL) as a low-grade lymphoproliferative neoplasm with ≥5 × 10^9^/L clonal B-cells in the peripheral circulation that express CD5, CD19, dimCD20, and CD23^1^. All cases of CLL are preceded by its pre-malignant counterpart called monoclonal B-cell lymphocytosis (MBL; defined as <5 × 10^9^/L clonal B-cells in the absence of lymphadenopathy, organomegaly, and cytopenias)^2^. MBL can be detected in ~5% of healthy individuals >40 years of age, making it one of the most common premalignant conditions in humans. Several groups have reported the risk of progression from MBL to CLL requiring therapy is ~1–2% per year^3,4^. Individuals with MBL and early-stage asymptomatic CLL who do not meet the 2018 International Workshop on CLL [IWCLL] criteria to initiate therapy should be offered close follow-up (“wait and watch”)^5^. The introduction of chemoimmunotherapy (such as fludarabine, cyclophosphamide, and rituximab [FCR]), and bendamustine and rituximab [BR]^6,7^, and more recently novel agents such as ibrutinib (Bruton tyrosine kinase inhibitor)^8^, idelalisib (phosphatidylinositol-3-kinase δ inhibitor)^9^, and venetoclax (BCL-2 inhibitor)^10^ has revolutionized the management of CLL. This review will focus on the current approach to risk stratification and management of patients with CLL. Although this schema can be used for CLL patients worldwide, the limited availability of many novel agents outside the US may limit its broader applicability.

## Contemporary risk stratification in CLL

There is a plethora of prognostic markers that help risk stratify CLL patients. Since the initial description of the Rai^11^ and Binet^12^ staging systems more than 4 decades ago, there have been tremendous advances in our understanding of the prognostic factors that predict time to first therapy and overall survival (OS) in CLL. These factors include simple laboratory tests (such as lymphocyte doubling time and lactate dehydrogenase), serum-based tests (such as beta-2 microglobulin and thymidine kinase)^13^, and flow-cytometry based tests (such as expression of CD38, ZAP-70, and CD49d)^14,15^. In 1999, two independent groups reported that CLL patients with higher degrees of somatic mutation in the immunoglobulin heavy chain variable gene (*IGHV*) experienced longer OS^16,17^. Patients with mutated *IGHV* (defined as a greater than 2% difference from the germline sequence) had a median OS of >20 years compared to those with unmutated *IGHV* who had median OS of 8 years. Although G-banding techniques detected chromosomal abnormalities in ~50% patients with CLL^18^, the introduction of FISH to evaluate for cytogenetic defects in non-dividing cells led Dohner and colleagues to propose a new prognostic model in CLL. Using a hierarchical classification scheme, they demonstrated that patients with del17p13 had the shortest OS (~3 years), followed by patients with del11q23 (~6.5 years), trisomy 12 (~9.2 years), negative FISH (~9.5 years), and del13q14 (~11 years)^19^. Somatic mutations detected by next-generation sequencing showed that genes involved in DNA damage and cell cycle control (*ATM, TP53, RB1, BIRC3*), Notch signaling (*NOTCH1, NOTCH2, FBXW7*), cytokine signaling (NRAS, KRAS, BRAF), inflammatory pathways (*MYD88, DDX3X, MAPK1*), and spliceosome machinery (*SF3B1*) were recurrently mutated in CLL^20^. Although the individual role of each of these genes in the pathogenesis and outcomes of CLL is currently being investigated, there is convincing data to show that CLL patients who harbor *NOTCH1* mutation (mostly occurring in patients with trisomy 12), *SF3B1* mutation (mostly occurring in patients with del13q14), and those with *TP53* mutation experience a shorter time to first therapy, progression-free survival (PFS), and OS^20–25^. In addition to these standard tests, other factors such as detection of subclonal mutations, micro-RNA signatures, B-cell receptor stereotypy, *IGHV* gene family usage, telomere length, and many others have been shown to provide important information about time to first therapy and OS in CLL patients^26^.

Since these prognostic markers may offer a discrepant prognosis in the same patient (i.e., some suggest a shorter time to first therapy and OS whereas others do not), many attempts have been made to integrate these into a combined risk score^27–29^. The most recent effort—the CLL International Prognostic Index (CLL-IPI)—studied ~28 prognostic variables among ~3400 patients treated on clinical trials across the world, and was validated in two independent cohorts of patients, including from Mayo Clinic and the Scandinavian CLL cohort^30^. Five factors were independently found to be associated with OS, including age >65 years, Rai stage I–IV, serum beta-2 microglobulin >3.5 mg/L, unmutated *IGHV* genes, and del17p by FISH or *TP53* mutation (Table 1). Four risk groups (low, intermediate, high, and very-high risk) with different 5-year OS (93%, 79%, 63%, and 23%, respectively) were identified. There are several limitations to the CLL-IPI: (a) it does not include patients treated on novel agents; (b) it does not include other important prognostic variables such as somatic genetic mutations detected by next-generation sequencing and patient comorbidities; and (c) although the CLL-IPI was primarily developed to predict OS, it is generally applied in predicting time to first therapy in newly diagnosed previously untreated CLL patients (given the rapid adoption of novel agents in the management of CLL). The 5-year treatment-free survival in the Mayo validation cohort of previously untreated patients in the low, intermediate, high, and very-high risk CLL-IPI groups was 78%, 54%, 32%, and 0%, respectively. Other studies have also confirmed the ability of the CLL-IPI risk score in predicting time to first therapy in previously untreated CLL patients^31–33^.

**Table 1:** The CLL-International Prognostic Index^30^

Prognostic factor	Points	
Del17p on FISH or *TP53* mutation	4	
Unmutated *IGHV* genes	2	
Serum β2 microglobulin >3.5 mg/L	2	
Rai stage I–IV	1	
Age >65 years	1	

Cumulative CLL-IPI score	Risk category	5-year TFS^a^
0–1	Low risk	78%
2–3	Intermediate risk	54%
4–6	High risk	32%
7–10	Very high risk	0%

*FISH* fluorescence in situ hybridization, *IGHV* immunoglobulin heavy chain gene, *TFS* treatment-free survival

^a^For the Mayo validation cohort

Minimal residual disease (MRD) at the end of CLL therapy is a powerful prognostic tool that predicts time to next therapy and OS in many studies, both in the chemoimmunotherapy and the novel agent era^34–36^. The 2018 IWCLL guidelines suggest using either multiparameter flow cytometry or allele-specific oligonucleotide polymerase chain reaction to detect MRD at 0.01% level (i.e., 1 leukemic cell in 10,000 leukocytes). An important limitation of using MRD as a biomarker in all CLL patients is that it cannot be used to predict outcomes in patients who are in the “wait and watch” asymptomatic phase of their disease.

## Management of previously untreated CLL

The vast majority of CLL patients have early-stage asymptomatic disease at diagnosis. Only those patients who meet the 2018 IWCLL criteria^5^ for initiation of therapy (Table 2) should be offered treatment.

**Table 2:** Updated 2018 International Workshop on CLL (IWCLL) guidelines to initiate CLL therapy^5^

Any **one** of the following criteria should be met to initiate CLL therapy:
• Progressive marrow failure, hemoglobin <10 gm/dL or platelet count of <100 × 10^9^/L
• Massive (≥6 cm below the left costal margin) or progressive or symptomatic splenomegaly
• Massive (≥10 cm in longest diameter) or progressive or symptomatic lymphadenopathy
• Progressive lymphocytosis with an increase of ≥50% over a 2-month period or lymphocyte doubling time of <6 months
• Autoimmune complications of CLL, that are poorly responsive to corticosteroids
• Symptomatic extranodal involvement (e.g., skin, kidney, lung, spine)
• Disease-related symptoms, including:
◦ Unintentional weight loss of ≥10% within the previous 6 months
◦ Significant fatigue
◦ Fever ≥38 °C for 2 or more weeks without evidence of infection
◦ Night sweats for ≥1 month without evidence of infection

### Patients who do **not** meet the 2018 IWCLL criteria for therapy

**Fig. 1:**
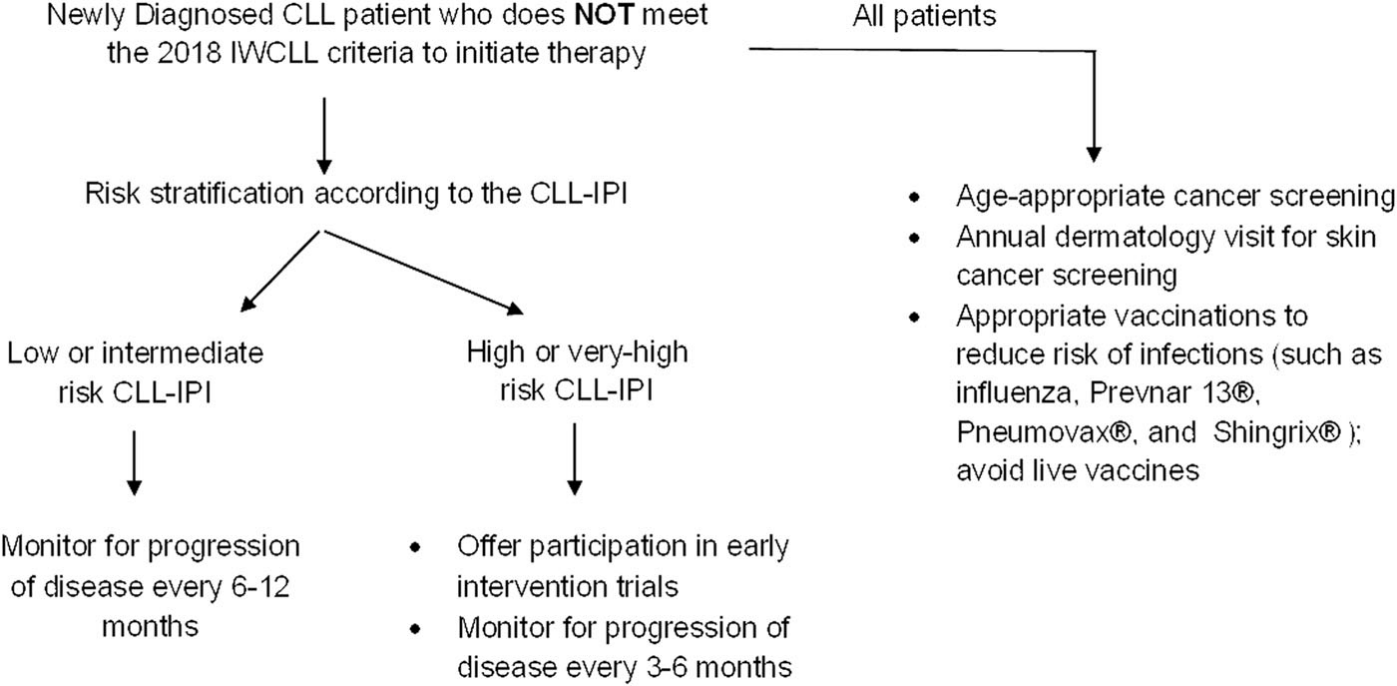
Suggested approach to the management of patients with newly diagnosed CLL who do not meet the 2018 IWCLL criteria for therapy

Figure 1 shows a suggested approach to the management of patients who do not meet the 2018 IWCLL criteria for therapy. All patients should undergo risk stratification according to the CLL-IPI at the time of diagnosis. Patients in the low- and intermediate-risk category CLL-IPI (~75% patients, median time to first therapy ~7 years) should be monitored for disease progression every 6–12 months. Patients in the high- and very high-risk CLL-IPI group (~25% patients, median time to first therapy ~2 years) should be monitored for disease progression every 3–6 months^30^. Regardless of the CLL-IPI score, all patients should be counseled for (a) increased risk of infections; with special attention to appropriate vaccinations according to the Centers for Disease Control and Prevention (CDC) guidelines^37^; (b) increased risk of non-hematologic malignancy, and recommendations to follow age-appropriate cancer screening; and (c) increased risk of skin cancers, with yearly full body skin exam by dermatology^38,39^. Patients with high- and very high-risk CLL may be offered treatment in early intervention clinical trials. The German CLL study group is conducting a phase 3 study (CLL12 trial) that randomizes patients with asymptomatic high-risk disease to ibrutinib vs. placebo; efficacy results from this trial are not available yet^40^. An important caveat to performing prognostic testing in *all* patients with newly diagnosed early-stage CLL is that this information may not be necessarily helpful in patients with advanced age, poor performance status, multiple co-morbidities, or those with limited life expectancy. Therefore, practicing oncologists must exercise their clinical judgment in obtaining these tests in newly diagnosed CLL patients who do not meet indications for therapy.

### Patients who meet the 2018 IWCLL criteria to start therapy

**Fig. 2:**
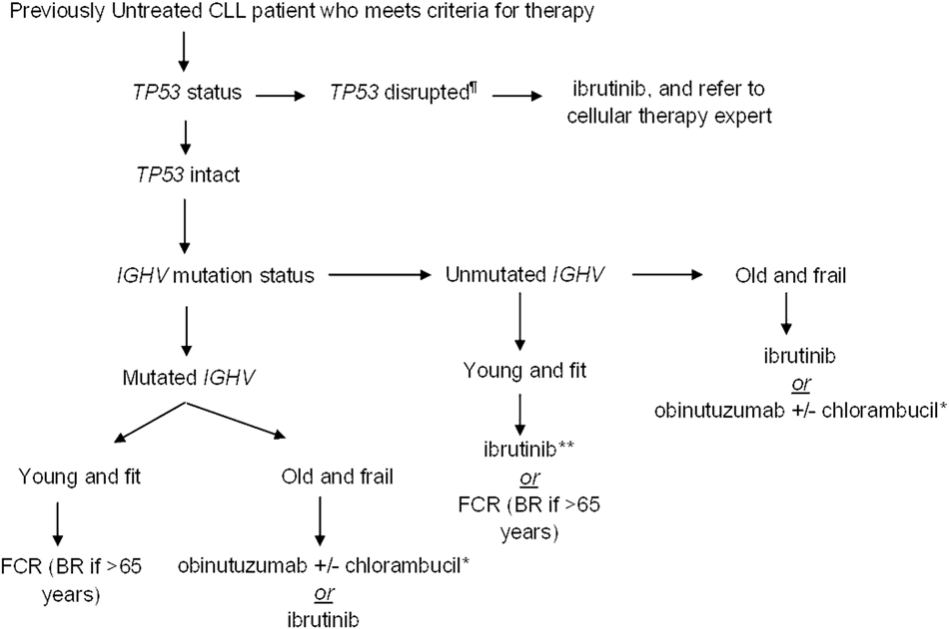
Suggested approach to the management of patients with previously untreated CLL who meet the 2018 IWCLL criteria for therapy (outside the context of clinical trials)

It should be noted that all patients who meet the 2018 IWCLL criteria should be offered therapy, regardless of their CLL-IPI risk group assignment. When feasible, all patients should be offered participation in well-designed clinical trials. Figure 2 shows a suggested approach for the management of previously untreated CLL patients, outside the context of clinical trials.

#### Ascertain the *TP53* status prior to therapy

The *TP53* status is one of the most important prognostic and predictive biomarkers in CLL. This should be ascertained using (a) CLL FISH panel to look for evidence of del17p13 and (b) Sanger sequencing or next-generation sequencing panel to evaluate for *TP53* mutations, with a cutoff of at least 10%. It is important to obtain both these tests, since ~3–5% patients will harbor a deleterious *TP53* mutation on DNA sequencing in the absence of del17p13 on CLL FISH; and multiple studies have shown these patients have equally poor outcomes^19,41–43^. Patients with *TP53* disruption have a short PFS and OS when treated with standard chemoimmunotherapy regimens such as FCR and BR, and are therefore not recommended for these patients^7,44^. In contrast, a single-arm phase 2 study of ibrutinib in previously untreated CLL patients with *TP53* disruption showed the overall response rate (ORR) was 97%, the cumulative incidence of progression at 2-years was 9%, and the estimated 2-year OS was 84%^45^. The unprecedented response rates and excellent outcomes make ibrutinib the treatment of choice in this group of patients. All patients with *TP53* mutation who need treatment should be referred to a cellular therapy expert, to discuss potential treatment options with either an allogeneic stem cell transplant or chimeric antigen receptor T-cell (CAR-T) therapy.

#### Ascertain the *IGHV* mutation status and fitness of the patient

Several studies have shown that *IGHV* mutated patients who receive FCR experience a long PFS (that can exceed 10 years)—making this an important treatment choice in patients who have no evidence of *TP53* disruption^46–48^. Treatment with FCR is recommended only in young (generally <65 years) and otherwise fit patients who are able to tolerate this intensive regimen. The CLL8 trial (phase 3 study comparing FCR to fludarabine and cyclophosphamide in previously untreated CLL) showed the ORR was 90% (complete response [CR] in 40% patients), and the median PFS was 52 months (the median PFS was not reached for the subset of *IGHV* mutated patients after a median follow-up of ~6 years)^44,46^. Long-term follow-up of the MDACC phase 2 FCR data show that after a median follow-up of ~13 years, the risk of secondary myeloid neoplasms was ~5% and of Richter’s transformation was ~8%^47^. A retrospective study from MD Anderson Cancer Center reported that patients who are MRD negative in the bone marrow at the end of 3 cycles of FCR therapy have similar long-term outcomes compared to patients who receive 6 cycles of therapy, suggesting it may be possible to avoid the cumulative toxicity that is frequently seen with this regimen^34^. The CLL10 trial (phase 3 trial comparing FCR to bendamustine and rituximab in previously untreated CLL) showed that therapy with FCR was associated with a higher CR rate (40% vs. 31%, *p* = 0.03) and a longer PFS compared to BR (55 months vs. 42 months, *p* < 0.001); although in the subset of patients >65 years of age, FCR led to more infectious and hematologic toxicities^7^.

For patients who are older and frail or have comorbidities (cumulative rating illness score [CIRS] of ≥7), therapy with chlorambucil and obinutuzumab (a humanized type II CD20 monoclonal antibody with a glycoengineered Fc domain) showed higher ORR (78% vs. 65%, *p* < 0.001) and longer PFS (median 29 months vs. 15 months, *p* < 0.001) compared to chlorambucil and rituximab in the CLL11 trial^49^. Since chlorambucil is frequently associated with significant myelosuppression and gastrointestinal toxicity, obinutuzumab as a single-agent may be used without loss of efficacy^50^. The RESONATE-2 trial compared ibrutinib to chlorambucil in elderly CLL patients (≥65 years; 69% CIRS score >6, del17p patients excluded), and showed that after a median follow-up of ~18 months, the estimated median PFS was not reached in the ibrutinib arm compared to 18.9 months in the chlorambucil arm. Ibrutinib also improved OS significantly; the estimated 24-month OS in the ibrutinib arm was 98% compared to 85% in the chlorambucil arm (despite crossover). The improvement in PFS and OS was similar among *IGHV* mutated and unmutated patients, indicating that ibrutinib was able to ameliorate the inferior outcomes of chemoimmunotherapy treated patients with unmutated *IGHV* genes^51^.

Two major phase 3 intergroup studies (ECOG 1912 [comparing FCR to ibrutinib and rituximab] and the ALLIANCE 041202 [comparing BR to ibrutinib and ibrutinib/rituximab]), and the CLL 14 trial (comparing venetoclax/obinutuzumab to chlorambucil/obinutuzumab) have completed accrual, and results from these trials will inform future practice in the frontline setting. Until the results of these trials are available, chemoimmunotherapy with FCR (<65 years) or BR (≥65 years) may be appropriate for young fit patients who have mutated *IGHV* genes. Among young fit patients with unmutated *IGHV* genes, ibrutinib is preferred in patients with an unfavorable FISH profile (such as del11q), whereas FCR and BR can be used in patients with standard risk FISH (del13q, normal and +12). Although both ibrutinib and obinutuzumab (with or without chlorambucil) may be used in old frail patients regardless of their *IGHV* mutation status, ibrutinib is preferred among those with unmutated *IGHV* genes. Patient preference plays a very important role in choosing therapy, where some patients prefer time-limited chemoimmunotherapy compared to daily oral continuous therapy with novel agents. Also, many patients have significant out of pocket costs for novel oral agents (financial toxicity), whereas infusional therapy may be completely covered by their insurance, making patients choose one type of treatment over another. A final recommendation regarding therapy should take into consideration all of these issues.

Unlike other chronic lymphoproliferative neoplasms, there is a limited role of maintenance therapy in CLL. Compared to placebo, maintenance therapy with lenalidomide prolonged PFS (but not OS) after both first-line and subsequent-line chemoimmunotherapy in CLL^52,53^. However, these trials enrolled high-risk patients (such as those with unmutated *IGHV* genes and high-risk cytogenetics by FISH), who would be treated with novel agents to begin with, making these results less pertinent in the current era.

## Treatment of relapsed/refractory CLL

All patients who have relapsed CLL should undergo a comprehensive assessment of their disease status, including a bone marrow aspirate and biopsy, and a CT scan of the chest, abdomen, and pelvis (a positron emission tomography [PET] scan is preferred if there is suspicion for Richter’s transformation). Also, all patients should have a CLL FISH panel and at minimum *TP53* mutation status re-analyzed prior to starting therapy. Although the *TP53* disruption status may not impact treatment choice given that all novel agents have excellent efficacy in this group of patients, the frequency of follow-up, monitoring for progression of disease, and the anticipated PFS benefit will be different in patients with *TP53* disruption compared to patients with intact *TP53*.

**Fig. 3:**
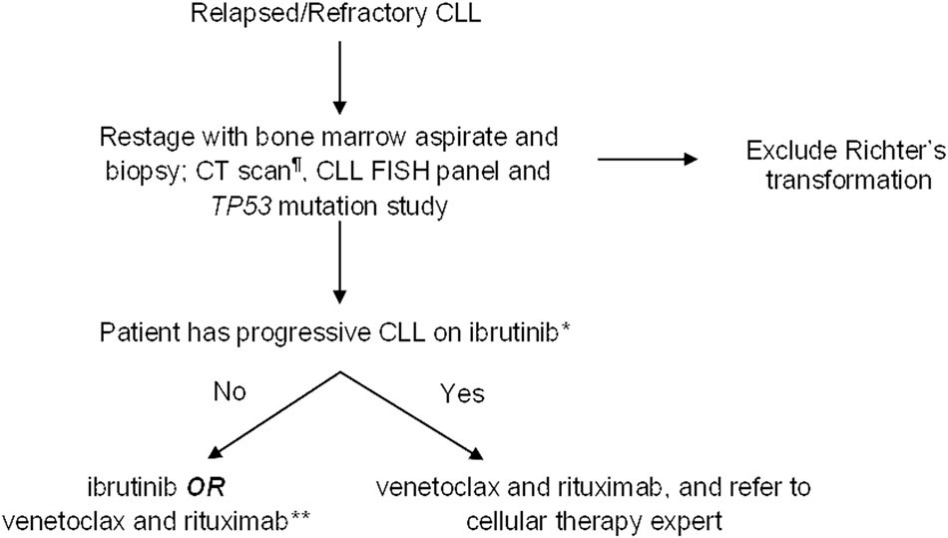
Suggested approach to the management of patients with relapsed/refractory CLL (outside the context of clinical trials)

Chemoimmunotherapy plays a less important role in the contemporary management of relapsed/refractory CLL. Circumstances where chemoimmunotherapy may be preferred include: patient preference, prohibitive cost of the novel agents, significant comorbidities that preclude the use of novel agents or if the patient has had a long (>3–5 years) remission duration after the first therapy. Several trials have compared the combination of a novel agent and chemoimmunotherapy to chemoimmunotherapy alone (such as ibrutinib/BR compared to BR [HELIOS]^54^, idelalisib/BR compared to BR)^55^ in relapsed/refractory CLL. Unfortunately, these studies lack the novel agent alone as a comparator arm, and therefore it is difficult to draw conclusions for routine practice from these studies. Figure 3 shows a suggested approach to the management of relapsed/refractory CLL, outside the context of clinical trials.

### Relapsed/refractory CLL that is naive to ibrutinib

Multiple clinical trials have confirmed the efficacy and safety of ibrutinib in relapsed CLL, among patients with and without *TP53* disruption. The pivotal RESONATE trial compared ibrutinib to ofatumumab in 391 patients with relapsed CLL (median number of prior therapies = 3). After a median follow-up of 9.4 months, the median PFS was not reached in the ibrutinib arm compared to 8.1 months in the ofatumumab arm (*p* < 0.001). Ibrutinib also significantly improved OS (12-month OS: 90% for ibrutinib vs. 81% for ofatumumab, hazard ratio for death, 0.43; *p* = 0.005). Similar results were seen among ibrutinib-treated patients with del17p13 and purine nucleoside analog refractory disease, who historically have poor outcomes^56^. Extended follow-up of the RESONATE study (median 19 months) showed that the estimated PFS at 24 months in the ibrutinib arm was 74%^57^. Five-year follow-up data from the single-arm phase 2 PCYC-1102/1103 study in which 101 relapsed/refractory CLL patients were treated with ibrutinib, the 5-year PFS was 44% (the median PFS by cytogenetic group according to FISH was 26 months for del17p13; 51 months for del11q23; and not reached for the other groups)^58^. A randomized study comparing ibrutinib to the combination of ibrutinib and rituximab in both previously untreated and relapsed/refractory CLL failed to show any incremental benefit of the addition of rituximab (2-year PFS in both groups was ~90%); suggesting that until more data become available, single-agent ibrutinib should be used in all patients^59^. The only exception may be in patients where a rapid response to treatment is desired, since the median time to normalization of the absolute lymphocyte count and MRD negative complete remission was shorter in the ibrutinib/rituximab arm.

The most common non-hematologic toxicities (≥grade 3) reported in the ibrutinib arm of RESONATE trial include pneumonia (10%), diarrhea (5%), fatigue (4%), and arthralgia (2%). Other toxicities include hypertension (~15%), atrial fibrillation (~10%), and major bleeding (~7%)^56^. Additionally, with longer follow-up, other adverse events, such as disseminated zoster^60^, *Pneumocystis jirovecci* pneumonia^61^ and invasive aspergillosis^62^ have been reported with ibrutinib use. In contrast to patients treated on clinical trials, data from patients treated in the context of routine clinical practice show that ~40% patients discontinue ibrutinib after ~2 years on therapy; the vast majority due to toxicity/intolerance^63,64^. For a detailed discussion of the practical considerations about patients receiving ibrutinib therapy, I would refer the reader to these excellent articles published recently^65,66^. An integrated analysis of ~300 CLL patients treated with ibrutinib at The Ohio State University showed that younger age, complex karyotype, and del17p13 were most commonly associated with CLL progression^67^.

Venetoclax has shown promising efficacy in the treatment of patients with relapsed CLL. In a phase 1b/2 study of venetoclax in relapsed/refractory CLL (*n* = 116, median number of prior therapies = 4), the ORR was 82%, and the estimated 15-month PFS was ~70% (at the recommended phase 2 dose level of 400 mg daily)^10^. In another study of single-agent venetoclax in 107 relapsed CLL patients with del17p13, the ORR was 79% and the estimated 12-month PFS was 72%^68^. On the basis of these results, venetoclax was Food and Drug Administration (FDA) approved for the treatment of relapsed/refractory CLL with del17p13. The MURANO study is a large phase 3 trial that compared venetoclax/rituximab to bendamustine/rituximab in patients with relapsed CLL (*n* = 389, including del17p13). After a median follow-up of ~2 years, the 2-year PFS was 85% in the venetoclax/rituximab arm compared to 36% in the bendamustine/rituximab arm (HR for progression or death: 0.17, *p* < 0.001). These results were consistent across patient subgroups; additionally, the rates of MRD negative status in the peripheral blood were significantly higher in the venetoclax/rituximab arm compared to bendamustine/rituximab (83% vs. 23%), suggesting that combination therapy with venetoclax and rituximab can achieve deep remissions even in patients with high-risk features^36^.

The most common side-effects of venetoclax are neutropenia (occurring in ~50% patients, which can be mitigated with growth factor support); tumor lysis syndrome (for which a gradual ramp-up dosing schema should be followed as specified in the package insert); and serious infections. In an analysis of 67 patients with relapsed CLL treated with single-agent venetoclax, fludarabine refractoriness and complex karyotype were associated with progression, whereas del17p13 or *TP53* mutations were not^69^. Longer follow-up with larger cohorts of patients will be necessary to determine which patients are most likely to experience disease progression on venetoclax.

Single-agent venetoclax is approved by the FDA for patients with relapsed/refractory CLL who have del17p by FISH. The combination of venetoclax and rituximab was recently approved by the FDA for *all* patients with relapsed/refractory CLL (regardless of the CLL FISH findings). However, given the lack of randomized trials comparing ibrutinib to venetoclax, the initial choice between these agents in relapsed CLL depends on patient comorbidities. In patients who have significant cardiovascular disease, are receiving anticoagulant therapy or have a high risk of bleeding, venetoclax may be more appropriate than ibrutinib. On the other hand, patients with significant tumor bulk (>10 cm lymph nodes) are at significant risk for developing tumor lysis syndrome with venetoclax, and ibrutinib may be more appropriate for this group of patients. It should be noted that these considerations for choosing ibrutinib vs. venetoclax are not absolute, and practicing oncologists may choose one over the other. Cross trial comparisons indicate patients who are treated with venetoclax (particularly in combination with rituximab) may achieve MRD negative complete remission sooner than those treated with ibrutinib as a single-agent, which would allow for patients to discontinue therapy. However, at this time, the data regarding discontinuation of novel agents in CLL patients who have achieved MRD negative complete remission are non-existent, and should therefore not impact the decision to start one type of treatment over another. Data regarding acquired resistance to ibrutinib (with the emergence of mutations in *BTK* and *PLCγ2* genes)^67^ and venetoclax (mutations in *BTG1* and homozygous deletions in *CDKN2A/B*)^70^ will allow earlier detection of sub-clinical relapse, and more targeted interventions to improve outcomes in patients with relapsed disease.

The combination of idelalisib and rituximab is also approved for the treatment of relapsed CLL^9^. However, idelalisib is associated with ≥grade 3 toxicity including colitis, transaminitis, and pneumonitis, in addition to infectious complications such as reactivation of cytomegalovirus and *P. jirovecii* pneumonia, making it less attractive if other options are available. Other novel agents with ongoing phase 3 trials in relapsed CLL include acalabrutinib, duvelisib, and umbralisib, among others; however, these are not approved for relapsed CLL at this time. Finally, many exciting combination studies of novel agents and monoclonal antibodies (doublet or triplet regimens) are currently being evaluated in relapsed CLL to achieve higher MRD negative complete remissions with the goal of discontinuing therapy, and results of these trials are eagerly awaited.

### Relapsed/refractory CLL progressing on ibrutinib

CLL patients who have progressive disease on ibrutinib represent a significant challenge, since many have rapid progression of the disease and experience poor outcomes^71^. It is critical to distinguish Richter’s transformation from progressive CLL in these patients. In a comprehensive analysis of risk factors for progression among ~300 ibrutinib-treated CLL patients, complex karyotype and BCL-6 abnormality were associated with Richter’s transformation^67^. More importantly, Richter’s transformation typically occurred in the first year whereas CLL progression events occurred in the second year of ibrutinib therapy. Unfortunately, the predictive ability of a PET scan in distinguishing Richter’s transformation from CLL progression in the era of novel agents is not very clear. In an analysis of 167 CLL patient progressing on BTK inhibitors, a standardized uptake value (SUV) cut-off of 10 was associated with poor sensitivity and specificity in predicting Richter’s transformation^72^. Therefore, all patients with rapidly proliferating disease (particularly in the first year on ibrutinib therapy), along with a high lactate dehydrogenase, should undergo a PET scan and an excisional or core needle biopsy of the most PET avid lymph node to ascertain Richter’s transformation.

In an analysis of 91 patients who were either refractory to or had intolerance to ibrutinib therapy, venetoclax was associated with an ORR of 65% (9% CR rate) and a median time to progression of ~24 months^73^. Data from a large cohort of CLL patients who were treated in routine clinical practice (i.e., outside the context of a clinical trial) also support the use of venetoclax in patients who progress on or have intolerance to ibrutinib, as opposed to using idelalisib and rituximab^74^. Unfortunately, given the low CR rate with single-agent venetoclax, it will be necessary to use combination strategies with either an anti-CD20 monoclonal antibody therapy or other novel agents. Many patients experience rapid disease progression when ibrutinib is stopped (since the dose ramp up to the approved daily therapeutic dose of 400 mg venetoclax takes ~5 weeks). Although no formal guidelines are available in such a situation, two possible approaches seem reasonable: (a) follow an accelerated ramp up dosing schedule of venetoclax (20 mg on day 1, 50 mg daily on days 2 and 3, 100 mg daily on days 4–7, 200 mg daily on days 8–14, and then 400 mg daily); or (b) continue with ibrutinib until the target dose of venetoclax is reached.

CAR-T cell therapy has also shown remarkable efficacy in the management of CLL patients who have disease progression on ibrutinib. In a study of 27 relapsed/refractory CLL patients (19 of whom had progression of disease on ibrutinib), anti-CD19 CAR-T cell therapy was associated with an ORR of 74% at 4 weeks, with a CR rate of 21%. Twenty patients (83%) developed cytokine release syndrome and eight (33%) developed neurotoxicity (which was reversible in all but one patient who had a fatal outcome). The median PFS was ~9 months and the median OS was not reached—none of the responding patients proceeded to an allogeneic stem cell transplant^75^. Although these results are encouraging, anti-CD19 CAR-T cell therapy is not approved for relapsed CLL yet, and therefore it is challenging to use this approach outside the context of clinical trials.

## Special situations

Richter’s transformation is the development of an aggressive B-cell neoplasm in CLL patients (the most common histology is diffuse large B-cell lymphoma (DLBCL) followed by Hodgkin lymphoma)^1^. A comprehensive review of the risk factors and management is outside the scope of this article; I would refer the readers to an excellent review published recently^76^. Among patients who develop DLBCL, establishing the clonal relationship between the DLBCL and CLL clone has important prognostic implications (patients with clonally unrelated DLBCL have much better outcomes than those that are clonally related)^77,78^. Among patients with Richter’s transformation who have not received prior CLL therapy or are clonally unrelated, multi-agent cytotoxic therapy with either (rituximab, cyclophosphamide, doxorubicin, vincristine, and prednisone [R-CHOP]) or infusional dose-adjusted etoposide, doxorubicin, and cyclophosphamide with vincristine, prednisone, and rituximab (DA-EPOCH-R) may be appropriate first-line therapy. For patients who develop transformation after ibrutinib or among patients with clonally related DLBCL where there is evidence of *TP53* disruption or *NOTCH1* mutation, PD1 inhibitor therapy with pembrolizumab and nivolumab has shown promising results^79,80^. Consolidation with an allogeneic SCT (or autologous SCT in patients where no matched donor is available) has shown to improve outcomes in this difficult-to-treat complication of CLL^81^. Therapy with adriamycin, bleomycin, vinblastine, and dacarbazine (ABVD) remains the standard of care for patients with Hodgkin transformation of CLL. Although the outcomes of Hodgkin transformation in CLL are better than patients who develop DLBCL transformation, these patients do significantly worse compared to those with de novo Hodgkin lymphoma^82^.

Autoimmune cytopenias occur in ~5–10% CLL patients (most commonly autoimmune hemolytic anemia [AIHA] and immune thrombocytopenia)^83^. In the absence of significant CLL tumor burden, these should be treated with corticosteroids or single-agent rituximab. In patients where CLL therapy is indicated or in whom the autoimmune cytopenias are refractory to first-line therapy, the general approach to the management of CLL patients as outlined above should be followed. Fludarabine (as a single-agent) can exacerbate hemolytic anemia in CLL and should be avoided in patients with a history of AIHA. FCR should be used with caution in CLL patients with a history of AIHA, although this remains a controversial issue. There is limited information on the use of ibrutinib in the treatment of autoimmune cytopenias in CLL (since the vast majority of clinical trials excluded patients with uncontrolled autoimmune cytopenias), although there is emerging evidence that it may be safe to do so^84,85^.

## Conclusion

There has been substantial progress in the management of CLL patients in the past decade. With the introduction of novel agents such as ibrutinib, idelalisib, and venetoclax, the role of chemoimmunotherapy in the treatment of CLL is being re-examined in the current era. Pressing questions in the next phase of CLL research include how best to combine novel agents, the sequencing of these treatments, and administering time-limited treatments to achieve deep remissions that allow stopping therapy.

## References

[1] Swerdlow, S. et al. (eds) *WHO Classification of Tumours of Haematopoietic and Lymphoid Tissues*, 4th edn (World Health Organization, 2008), Lyon, France.

[2] Swerdlow SH (2016). The 2016 revision of the World Health Organization classification of lymphoid neoplasms. Blood.

[3] Parikh Sameer A., Chaffee Kari G., Larson Melissa C., Hampel Paul J., Call Timothy G., Ding Wei, Kenderian Saad S., Leis Jose F., Chanan-Khan Asher A., Conte Michael J., Bowen Deborah, Schwager Susan M., Slager Susan L., Hanson Curtis A., Kay Neil E., Shanafelt Tait D. (2018). Outcomes of a large cohort of individuals with clinically ascertained high-count monoclonal B-cell lymphocytosis. Haematologica.

[4] Rawstron AC (2008). Monoclonal B-cell lymphocytosis and chronic lymphocytic leukemia. N. Engl. J. Med..

[5] Hallek Michael, Cheson Bruce D., Catovsky Daniel, Caligaris-Cappio Federico, Dighiero Guillermo, Döhner Hartmut, Hillmen Peter, Keating Michael, Montserrat Emili, Chiorazzi Nicholas, Stilgenbauer Stephan, Rai Kanti R., Byrd John C., Eichhorst Barbara, O’Brien Susan, Robak Tadeusz, Seymour John F., Kipps Thomas J. (2018). iwCLL guidelines for diagnosis, indications for treatment, response assessment, and supportive management of CLL. Blood.

[6] Keating MJ (2005). Early results of a chemoimmunotherapy regimen of fludarabine, cyclophosphamide, and rituximab as initial therapy for chronic lymphocytic leukemia. J. Clin. Oncol..

[7] Eichhorst B (2016). First-line chemoimmunotherapy with bendamustine and rituximab versus fludarabine, cyclophosphamide, and rituximab in patients with advanced chronic lymphocytic leukaemia (CLL10): an international, open-label, randomised, phase 3, non-inferiority trial. Lancet Oncol..

[8] Byrd JC (2013). Targeting BTK with ibrutinib in relapsed chronic lymphocytic leukemia. N. Engl. J. Med..

[9] Furman RR (2014). Idelalisib and rituximab in relapsed chronic lymphocytic leukemia. N. Engl. J. Med..

[10] Roberts AW (2016). Targeting BCL2 with venetoclax in relapsed chronic lymphocytic leukemia. N. Engl. J. Med..

[11] Rai KR (1975). Clinical staging of chronic lymphocytic leukemia. Blood.

[12] Binet JL (1981). A new prognostic classification of chronic lymphocytic leukemia derived from a multivariate survival analysis. Cancer.

[13] Hallek M (1996). Serum beta(2)-microglobulin and serum thymidine kinase are independent predictors of progression-free survival in chronic lymphocytic leukemia and immunocytoma. Leuk. Lymphoma.

[14] Rassenti LZ (2008). Relative value of ZAP-70, CD38, and immunoglobulin mutation status in predicting aggressive disease in chronic lymphocytic leukemia. Blood.

[15] Shanafelt TD (2008). CD49d expression is an independent predictor of overall survival in patients with chronic lymphocytic leukaemia: a prognostic parameter with therapeutic potential. Br. J. Haematol..

[16] Damle RN (1999). Ig V gene mutation status and CD38 expression as novel prognostic indicators in chronic lymphocytic leukemia. Blood.

[17] Hamblin TJ, Davis Z, Gardiner A, Oscier DG, Stevenson FK (1999). Unmutated Ig V(H) genes are associated with a more aggressive form of chronic lymphocytic leukemia. Blood.

[18] Juliusson G (1990). Prognostic subgroups in B-cell chronic lymphocytic leukemia defined by specific chromosomal abnormalities. N. Engl. J. Med..

[19] Dohner H (2000). Genomic aberrations and survival in chronic lymphocytic leukemia. N. Engl. J. Med..

[20] Wang L (2011). SF3B1 and other novel cancer genes in chronic lymphocytic leukemia. N. Engl. J. Med..

[21] Quesada V (2012). Exome sequencing identifies recurrent mutations of the splicing factor SF3B1 gene in chronic lymphocytic leukemia. Nat. Genet..

[22] Oscier DG (2013). The clinical significance of NOTCH1 and SF3B1 mutations in the UK LRF CLL4 trial. Blood.

[23] Rossi D (2012). Mutations of NOTCH1 are an independent predictor of survival in chronic lymphocytic leukemia. Blood.

[24] Sportoletti P (2010). NOTCH1 PEST domain mutation is an adverse prognostic factor in B-CLL. Br. J. Haematol..

[25] Rossi D (2013). Association between molecular lesions and specific B-cell receptor subsets in chronic lymphocytic leukemia. Blood.

[26] Parikh SA, Shanafelt TD (2016). Prognostic factors and risk stratification in chronic lymphocytic leukemia. Semin. Oncol..

[27] Wierda WG (2007). Prognostic nomogram and index for overall survival in previously untreated patients with chronic lymphocytic leukemia. Blood.

[28] Rossi D (2013). Integrated mutational and cytogenetic analysis identifies new prognostic subgroups in chronic lymphocytic leukemia. Blood.

[29] Pflug N (2014). Development of a comprehensive prognostic index for patients with chronic lymphocytic leukemia. Blood.

[30] CLL-IPI working group. An international prognostic index for patients with chronic lymphocytic leukaemia (CLL-IPI): a meta-analysis of individual patient data. *Lancet Oncol.* **17**, 779–790 (2016). 10.1016/S1470-2045(16)30029-8 27185642

[31] Molica S (2017). Assessing time to first treatment in early chronic lymphocytic leukemia (CLL): a comparative performance analysis of five prognostic models with inclusion of CLL-international prognostic index (CLL-IPI). Leuk. Lymphoma.

[32] Gentile M (2016). Validation of the CLL-IPI and comparison with the MDACC prognostic index in newly diagnosed patients. Blood.

[33] Munoz-Novas C (2018). The International Prognostic Index for patients with chronic lymphocytic leukemia has the higher value in predicting overall outcome compared with the Barcelona-Brno Biomarkers Only Prognostic Model and the MD Anderson Cancer Center Prognostic Index. Biomed. Res. Int..

[34] Strati P (2014). Eradication of bone marrow minimal residual disease may prompt early treatment discontinuation in CLL. Blood.

[35] Bottcher S (2012). Minimal residual disease quantification is an independent predictor of progression-free and overall survival in chronic lymphocytic leukemia: a multivariate analysis from the randomized GCLLSG CLL8 trial. J. Clin. Oncol..

[36] Seymour JF (2018). Venetoclax–rituximab in relapsed or refractory chronic lymphocytic leukemia. N. Engl. J. Med..

[37] *Recommended Immunization Schedule for Adults Aged 19 Years or Older, United States, 2018*. https://www.cdc.gov/vaccines/schedules/hcp/imz/adult.html#f5. Accessed April 26, 2018. 10.7326/M17-3439 29404596

[38] Mansfield AS (2014). Skin cancer surveillance and malignancies of the skin in a community-dwelling cohort of patients with newly diagnosed chronic lymphocytic leukemia. J. Oncol. Pract..

[39] Solomon B M, Chaffee K G, Moreira J, Schwager S M, Cerhan J R, Call T G, Kay N E, Slager S L, Shanafelt T D (2015). Risk of non-hematologic cancer in individuals with high-count monoclonal B-cell lymphocytosis. Leukemia.

[40] Langerbeins P (2015). The CLL12 trial protocol: a placebo-controlled double-blind Phase III study of ibrutinib in the treatment of early-stage chronic lymphocytic leukemia patients with risk of early disease progression. Future Oncol..

[41] Rossi D (2009). The prognostic value of TP53 mutations in chronic lymphocytic leukemia is independent of Del17p13: implications for overall survival and chemorefractoriness. Clin. Cancer Res..

[42] Zenz T (2008). Monoallelic TP53 inactivation is associated with poor prognosis in chronic lymphocytic leukemia: results from a detailed genetic characterization with long-term follow-up. Blood.

[43] Rossi D (2014). Clinical impact of small TP53 mutated subclones in chronic lymphocytic leukemia. Blood.

[44] Hallek M (2010). Addition of rituximab to fludarabine and cyclophosphamide in patients with chronic lymphocytic leukaemia: a randomised, open-label, phase 3 trial. Lancet.

[45] Farooqui MZ (2015). Ibrutinib for previously untreated and relapsed or refractory chronic lymphocytic leukaemia with TP53 aberrations: a phase 2, single-arm trial. Lancet Oncol..

[46] Fischer K (2016). Long-term remissions after FCR chemoimmunotherapy in previously untreated patients with CLL: updated results of the CLL8 trial. Blood.

[47] Thompson PA (2016). Fludarabine, cyclophosphamide, and rituximab treatment achieves long-term disease-free survival in IGHV-mutated chronic lymphocytic leukemia. Blood.

[48] Rossi D (2015). Molecular prediction of durable remission after first-line fludarabine-cyclophosphamide-rituximab in chronic lymphocytic leukemia. Blood.

[49] Goede V (2014). Obinutuzumab plus chlorambucil in patients with CLL and coexisting conditions. N. Engl. J. Med..

[50] Gay Nathan D., Kozin Eliana, Okada Craig, Danilov Alexey V., Spurgeon Stephen (2017). Obinutuzumab monotherapy in previously untreated chronic lymphocytic leukemia. Leukemia & Lymphoma.

[51] Burger JA (2015). Ibrutinib as initial therapy for patients with chronic lymphocytic leukemia. N. Engl. J. Med..

[52] Fink AM (2017). Lenalidomide maintenance after first-line therapy for high-risk chronic lymphocytic leukaemia (CLLM1): final results from a randomised, double-blind, phase 3 study. Lancet Haematol..

[53] Chanan-Khan AA (2017). Lenalidomide maintenance therapy in previously treated chronic lymphocytic leukaemia (CONTINUUM): a randomised, double-blind, placebo-controlled, phase 3 trial. Lancet Haematol..

[54] Chanan-Khan A (2016). Ibrutinib combined with bendamustine and rituximab compared with placebo, bendamustine, and rituximab for previously treated chronic lymphocytic leukaemia or small lymphocytic lymphoma (HELIOS): a randomised, double-blind, phase 3 study. Lancet Oncol..

[55] Zelenetz AD (2017). Idelalisib or placebo in combination with bendamustine and rituximab in patients with relapsed or refractory chronic lymphocytic leukaemia: interim results from a phase 3, randomised, double-blind, placebo-controlled trial. Lancet Oncol..

[56] Byrd JC (2014). Ibrutinib versus ofatumumab in previously treated chronic lymphoid leukemia. N. Engl. J. Med..

[57] Brown JR (2017). Extended follow-up and impact of high-risk prognostic factors from the phase 3 RESONATE study in patients with previously treated CLL/SLL. Leukemia.

[58] O’Brien S (2018). Single-agent ibrutinib in treatment-naïve and relapsed/refractory chronic lymphocytic leukemia: a 5-year experience. Blood.

[59] Burger JA (2017). Randomized trial of ibrutinib versus ibrutinib plus rituximab (Ib+R) in patients with chronic lymphocytic leukemia (CLL). Blood.

[60] Giridhar KV, Shanafelt T, Tosh PK, Parikh SA, Call TG (2017). Disseminated herpes zoster in chronic lymphocytic leukemia (CLL) patients treated with B-cell receptor pathway inhibitors. Leuk. Lymphoma.

[61] Ahn IE (2016). Atypical *Pneumocystis jirovecii* pneumonia in previously untreated patients with CLL on single-agent ibrutinib. Blood.

[62] Ghez D (2018). Early-onset invasive aspergillosis and other fungal infections in patients treated with ibrutinib. Blood.

[63] Mato Anthony R., Nabhan Chadhi, Thompson Meghan C., Lamanna Nicole, Brander Danielle M., Hill Brian, Howlett Christina, Skarbnik Alan, Cheson Bruce D., Zent Clive, Pu Jeffrey, Kiselev Pavel, Goy Andre, Claxton David, Isaac Krista, Kennard Kaitlin H., Timlin Colleen, Landsburg Daniel, Winter Allison, Nasta Sunita D., Bachow Spencer H., Schuster Stephen J., Dorsey Colleen, Svoboda Jakub, Barr Paul, Ujjani Chaitra S. (2018). Toxicities and outcomes of 616 ibrutinib-treated patients in the United States: a real-world analysis. Haematologica.

[64] Parikh SA (2015). Ibrutinib therapy for chronic lymphocytic leukemia (CLL): an analysis of a large cohort of patients treated in routine clinical practice. Blood.

[65] Brown JR (2018). How I treat CLL patients with ibrutinib. Blood.

[66] Gribben JG (2018). Optimising outcomes for patients with chronic lymphocytic leukaemia on ibrutinib therapy: European recommendations for clinical practice. Br. J. Haematol..

[67] Woyach JA (2017). BTKC481S-mediated resistance to ibrutinib in chronic lymphocytic leukemia. J. Clin. Oncol..

[68] Stilgenbauer S (2016). Venetoclax in relapsed or refractory chronic lymphocytic leukaemia with 17p deletion: a multicentre, open-label, phase 2 study. Lancet Oncol..

[69] Anderson MA (2017). Clinicopathological features and outcomes of progression of CLL on the BCL2 inhibitor venetoclax. Blood.

[70] Frenzel LP (2017). Mechanisms of venetoclax resistance in chronic lymphocytic leukemia. Blood.

[71] Jain P (2015). Outcomes of patients with chronic lymphocytic leukemia after discontinuing ibrutinib. Blood.

[72] Mato AR (2017). Analysis of PET-CT to identify Richter’s transformation in 167 patients with disease progression following kinase inhibitor therapy. Blood.

[73] Jones JA (2018). Venetoclax for chronic lymphocytic leukaemia progressing after ibrutinib: an interim analysis of a multicentre, open-label, phase 2 trial. Lancet Oncol..

[74] Mato A. R., Nabhan C., Barr P. M., Ujjani C. S., Hill B. T., Lamanna N., Skarbnik A. P., Howlett C., Pu J. J., Sehgal A. R., Strelec L. E., Vandegrift A., Fitzpatrick D. M., Zent C. S., Feldman T., Goy A., Claxton D. F., Bachow S. H., Kaur G., Svoboda J., Nasta S. D., Porter D., Landsburg D. J., Schuster S. J., Cheson B. D., Kiselev P., Evens A. M. (2016). Outcomes of CLL patients treated with sequential kinase inhibitor therapy: a real world experience. Blood.

[75] Turtle Cameron J., Hay Kevin A., Hanafi Laïla-Aïcha, Li Daniel, Cherian Sindhu, Chen Xueyan, Wood Brent, Lozanski Arletta, Byrd John C., Heimfeld Shelly, Riddell Stanley R., Maloney David G. (2017). Durable Molecular Remissions in Chronic Lymphocytic Leukemia Treated With CD19-Specific Chimeric Antigen Receptor–Modified T Cells After Failure of Ibrutinib. Journal of Clinical Oncology.

[76] Rossi D, Spina V, Gaidano G (2018). Biology and treatment of Richter syndrome. Blood.

[77] Parikh SA, Kay NE, Shanafelt TD (2014). How we treat Richter syndrome. Blood.

[78] Rossi D (2011). The genetics of Richter syndrome reveals disease heterogeneity and predicts survival after transformation. Blood.

[79] Ding W (2017). Pembrolizumab in patients with CLL and Richter transformation or with relapsed CLL. Blood.

[80] Jain N (2016). Nivolumab combined with ibrutinib for CLL and Richter transformation: a Phase II trial. Blood.

[81] Cwynarski K (2012). Autologous and allogeneic stem-cell transplantation for transformed chronic lymphocytic leukemia (Richter’s Syndrome): a retrospective analysis from the Chronic Lymphocytic Leukemia Subcommittee of the Chronic Leukemia Working Party and Lymphoma Working Party of the European Group for Blood and Marrow Transplantation. J. Clin. Oncol..

[82] Parikh SA (2015). Hodgkin transformation of chronic lymphocytic leukemia: Incidence, outcomes, and comparison to de novo Hodgkin lymphoma. Am. J. Hematol..

[83] Tsang M, Parikh SA (2017). A concise review of autoimmune cytopenias in chronic lymphocytic leukemia. Curr. Hematol. Malig. Rep..

[84] Hampel, P. J. et al. Autoimmune cytopenias in patients with chronic lymphocytic leukaemia treated with ibrutinib in routine clinical practice at an academic medical centre. *Br. J. Haematol.*10.1111/bjh.15545 (2018). 10.1111/bjh.15545 PMC6234062 30117139

[85] Vitale C (2016). Autoimmune cytopenias in patients with chronic lymphocytic leukemia treated with ibrutinib. Haematologica.

